# Identification of novel target genes in exaggerated cardiac remodeling following myocardial infarction in diabetes

**DOI:** 10.3389/fendo.2025.1536639

**Published:** 2025-03-14

**Authors:** Yanru Duan, Shihan Zhang, Yihua Xia, Huili Li, Demin Liu, Yunhui Du

**Affiliations:** ^1^ Beijing Anzhen Hospital, Capital Medical University, Beijing Institute of Heart, Lung and Blood Vessel Diseases, Beijing, China; ^2^ Medical Oncology Department, Pediatric Oncology Center, Beijing Children’s Hospital, Capital Medical University, National Center for Children’s Health, Beijing Key Laboratory of Pediatric Hematology Oncology, National Key Clinical Discipline of Pediatric Oncology, Key Laboratory of Major Diseases in Children, Ministry of Education, Beijing, China; ^3^ Department of Cardiology, China-Japan Friendship Hospital, Beijing, China; ^4^ Department of Anesthesiology, Beijing Anzhen Hospital, Capital Medical University, Beijing, China; ^5^ Emergency Department, The State Key Laboratory for Complex, Severe and Rare Diseases, Peking Union Medical College Hospital, Beijing, China; ^6^ Department of Cardiology, The Second Hospital of Hebei Medical University, Shijiazhuang, Hebei, China

**Keywords:** diabetes, myocardial infarction, lncRNA, mRNA, cardiomyocytes

## Abstract

**Introduction:**

Diabetes mellitus is a major risk factor for myocardial infarction (MI), yet its molecular mechanisms exacerbating post-MI cardiac remodeling remain unclear.

**Methods:**

Type 2 diabetes mellitus mouse model was developed through a high-sugar and high-fat diet (HFD), followed by MI surgery. Four weeks post-surgery, cardiac function was evaluated via echocardiography, and cardiac pathology was examined using Masson's trichrome and wheat germ agglutinin staining. High-throughput sequencing identified differentially expressed mRNAs and long non-coding RNAs (LncRNAs) in diabetic mice with MI. Gene Ontology (GO) and Kyoto Encyclopedia of Genes and Genomes (KEGG) pathway analyses, along with LncRNA-target-gene analysis, were performed. Validation in human samples of diabetic patients with STEMI confirmed the influence of HFD on the expression of specific genes.

**Results:**

The results demonstrate that diabetes significantly impairs cardiac function, exacerbates cardiac fibrosis and hypertrophy. In addition, our extensive examination of human samples has conclusively demonstrated that diabetes significantly modulates the expression of genes (Rapgef5 and Ing1) within the cardiac tissue of individuals afflicted with STEMI, underscoring the intricate interplay between these conditions. In addition, we have found that Rapgef5 and Ing1 are involved in diabetes-mediated cardiomyocyte apoptosis and proliferation following myocardial infarction.

**Discussion:**

Diabetes aggravates post-MI remodeling via Rapgef5/Ing1-mediated apoptosis and proliferation, these findings highlight novel therapeutic targets for diabetic cardiovascular complications.

## Introduction

Myocardial infarction (MI) represents a severe cardiovascular disease that poses a significant threat to public health (1). With an incidence rate of 8.5 million cases, and exacerbated by lifestyle changes, the prevalence of MI is escalating annually (2). Despite significant advancements in reperfusion therapy, the incidence and mortality rates of MI have not been effectively reduced (1), suggesting the involvement of other risk factors that significantly impact patient prognosis.

Diabetes mellitus, a well-established risk factor for MI, is a prevalent metabolic disorder primarily caused by defects in insulin secretion or action, leading to chronic hyperglycemia (3). Prolonged and uncontrolled hyperglycemia disrupts glucose and lipid metabolism, precipitating vascular pathologies and ultimately contributing to the onset of MI (4). Numerous clinical studies have demonstrated higher mortality rates among diabetic patients with MI compared to non-diabetic individuals (5). Elevated glucose levels are a hallmark feature that exacerbates myocardial remodeling post-MI (6). However, the transcriptomic mechanisms underlying diabetes-induced aggravation of post-MI cardiac remodeling remain elusive.

Long non-coding RNAs (LncRNAs), a class of non-protein-coding transcripts, regulate gene expression through intricate interactions with DNA, RNA, or proteins. They play pivotal roles in cellular differentiation, disease initiation, and progression (7). Notably, LncRNAs are indispensable in cardiovascular development and the pathogenesis of cardiovascular diseases, participating in processes such as cell proliferation, hypertrophy, and apoptosis (8). Their involvement is intimately linked to atherosclerosis (9), MI (10), heart failure (11), and other cardiovascular disorders (12). However, the expression profiles of LncRNAs and mRNAs in the context of diabetes-associated MI remain unclear, and whether LncRNAs contribute to the enhanced cardiac injury post-MI in diabetic patients is yet to be determined.

Thus, the current study aims to bridge this knowledge gap by conducting comprehensive genome-wide transcriptional profiling and LncRNA sequencing analyses. This will provide deeper insights into the mechanisms underlying how high-fat diet (HFD)-induced cardiac injury exacerbates myocardial infarction.

## Materials and methods

### Participants

Participants with ST-elevation myocardial infarction (STEMI) with or without diabetes, were recruited from Beijing Anzhen Hospital, Capital Medical University, Beijing, China. Inclusion Criteria: eligible participants were aged between 18 and 75 years. STEMI was diagnosed as characteristic symptoms lasting more than 30min, with electrocardiographic ST-segment elevation at least 0.1mV in>2 contiguous limb leads or at least 0.2 mV in > continuous precordial leads, 0.1 mV within 2 contiguous lime leads, and elevated creatine kinase elevation exceeding twice the upper limit of normal. Diabetes was defined as meeting any of the following criteria: fasting plasma glucose (FPG) ≥7.0 mmol/L, glycated hemoglobin (HbA1c) ≥6.5%, previously diagnosed DM with ongoing glucose-lowering therapy. The exclusion criteria were as follows: patients with severe heart failure, serious heart valve disease, genetic or confirmed cardiomyopathy, familial hypercholesterolemia, digestive diseases, current infectious diseases, chronic kidney diseases, or malignancies at baseline, Major surgery, trauma, cerebrovascular accidents, Type 1 diabetes mellitus, gestational diabetes (adjusted per study objectives), or use of glucose-lowering agents with established cardiovascular effects (e.g., SGLT2 inhibitors, GLP-1 receptor agonists). The clinical characteristics of the STEMI patients are summarized in **Supplementary Table S1**. The study was approved by the Medical Ethics Committee of Beijing Anzhen Hospital, and written informed consent was obtained from all participants. Heart tissue samples were collected at admission, immediately separated, and stored at −80°C until further analysis.

### Animal model

All experiments in this study were conducted in adherence to the NIH Guidelines on the Use of Laboratory Animals and were approved by the Research Ethics Committee of Capital Medical University. Male C57BL/6 mice, 8 weeks old and weighing 20 ± 2 grams, were used in this study. Wild-type (WT) mice were purchased from Beijing Si-Bei-Fu Experimental Animal Technology Co., Ltd. The mice were randomized to receive either a high-fat diet (HFD) (60% kcal fat, D12492i; Research Diets Inc.) or a standard diet (ND, D12450Bi) for 12 weeks to induce type 2 diabetes, as determined by fasting blood glucose levels. After 8 weeks, the mice were subjected to general anesthesia (isoflurane 2%), and their hearts were exposed via a small left thoracotomy. Ligation of the distal third of the left anterior descending artery (LAD) was performed using 6-0 silk sutures. The heart was then replaced within the intrathoracic space, followed by skin closure and manual pneumothorax evacuation for an additional four weeks.

### Echocardiography

The value used for analysis was the mean value over the three measured cardiac cycles. M-mode images of mice were acquired using a Visual Sonics Vevo 2100 system equipped with a 30 MHz transducer (MS-400; Visual Sonics). The mice were first weighed, anesthetized, and secured on an operating table. The probe angle was adjusted to obtain a long-axis view of the left ventricle. Subsequently, the probe was rotated 90°clockwise to capture the short-axis view of the left ventricle. The values used for analysis were averaged over three measured cardiac cycles.

### RNA preparation, transcriptome sequencing, and analysis

Total RNA was extracted from heart tissue using TRIzol reagent (Invitrogen, Carlsbad, Canada). The purification of RNA was performed with a NanoDrop 2000 microspectrophotometer. RNA integrity was assessed using an Agilent 2100 Bioanalyzer in conjunction with an Agilent RNA 6000 Nano Kit. After quantifying RNA concentration, a Small RNA Sample Pre-Kit was employed to construct a library from total RNA, utilizing the unique structural features of small RNAs at their 3′ and 5′ ends (the 5′ end possesses a complete phosphate group, while the 3′ end features a hydroxyl group). Adapters were ligated to both ends of the small RNAs, followed by reverse transcription to synthesize complementary DNA (cDNA). Target DNA fragments were then amplified via Polymerase Chain Reaction (PCR), and the resulting amplicons were separated by polyacrylamide gel electrophoresis (PAGE). The cDNA library was recovered by excising the relevant bands from the gel. Upon construction of the cDNA library, preliminary quantification was conducted using a Qubit 2.0 fluorometer. The cDNA was subsequently diluted to 1 ng/μl, and the insert size of the library was assessed with the Agilent 2100 Bioanalyzer. Once the insert size met our criteria, we applied quantitative PCR (qPCR) to determine the effective concentration of the library, ensuring that the library quality was satisfactory (with an effective concentration exceeding 2 nM). Following comprehensive quality control of the library, Illumina sequencing was performed using the HiSeq/MiSeq platform. A random variance model (RVM) t-test was applied to filter differentially expressed genes based on a specified p-value threshold. Hierarchical clustering was subsequently utilized to analyze the differentially expressed long non-coding RNAs (lncRNAs) and messenger RNAs (mRNAs).

### Bioinformatics analysis

Gene Ontology (GO) analysis, encompassing Biological Process (BP), Molecular Function (MF), and Cellular Component (CC) categories, was conducted to elucidate the functional roles of the differentially expressed long non-coding RNAs (lncRNAs) and messenger RNAs (mRNAs). Subsequently, pathway analysis was performed to identify the significant functional implications of the differential genes, utilizing the Kyoto Encyclopedia of Genes and Genomes (KEGG) database. In all analyses, we employed the two-sided Fisher’s exact test along with the Benjamini–Hochberg procedure to correct for multiple testing. Statistical significance thresholds were set at p < 0.01 for GO analyses and p < 0.05 for pathway analyses.

Based on gene (protein) interactions from the KEGG database, a global signal transduction network (Signal-net) was constructed to illustrate the interactions among differentially expressed genes in the treated groups. Visualization of the network was achieved using Cytoscape (version 3.6.0). In the network graph, nodes represent genes, while edges indicate relationships between them. The degree of a node is defined by the number of links it has to other nodes, with higher degrees indicating a more pivotal role within the Signal-net. This signal transduction network analysis serves as a method to identify core genes with significant regulatory potential over other genes.

A pathway network was constructed based on the KEGG pathway analysis, with visualization performed using Cytoscape (version 3.6.0). In this network graph, nodes represent pathways, while edges indicate the relationships among them. The degree of each node, defined by the number of connections to other nodes, signifies its centrality within the network; pathways with higher degrees are considered to hold more critical positions in the signal transduction network (Signal-net). Consequently, pathway network analysis serves as a method for identifying core pathways that possess significant regulatory capabilities over other pathways.

The lncRNA-mRNA co-expression network analysis was conducted to explore functional annotations. The networks were visualized using Cytoscape (version 3.6.0). In the lncRNA-target gene networks, genes are depicted as circles, lncRNAs as squares, and relationships as connecting lines. The degree of each lncRNA is determined by the number of genes it regulates, while the degree of each gene is influenced by the number of lncRNAs that regulate it. Those lncRNAs and genes with the highest degrees are identified as key components within the network.

### Real-time quantitative PCR

Total RNA was isolated using the Trizol reagent method (Invitrogen). This total RNA was subsequently utilized for first-strand complementary DNA (cDNA) synthesis. Quantitative reverse transcription PCR (qRT-PCR) was conducted with RT2 SYBR Green Mastermix (PARN-026Z, QIAGEN) on a 7500 Real-Time PCR system (Thermo Fisher Scientific, Inc.). The PCR conditions included an initial denaturation step at 95°C for 10 minutes, followed by 40 cycles of denaturation at 95°C for 15 seconds and annealing/extension at 60°C for 1 minute. Gene expression levels were quantified using the CT value, and fold changes in expression were calculated using the 2^^-ΔΔCT^ method. All samples were analyzed in triplicate, and primers for qPCR were obtained from Sangon Biotech (**Supplementary Table S2**).

### CCK8 assay

CCK-8 kit (Dojindo, Shanghai, China) was used to measure proliferation of AC-16 cells. 1,000 cells per well in a volume of 100 μl were cultured in ten replicate wells in a 96-well plate in medium containing 10% FBS. Then the CCK-8 reagent (10 μl) was added to 90 μl DMEM to generate a working solution, of which 100 μl was added per well and incubated for 1 h. The optical densities were measured at a spectral wavelength of 450nm using a microplate reader (Thermo Fisher Scientific, Inc.).

### Cell apoptosis assay

Cell apoptosis was quantitatively assessed via flow cytometry using a FITC-Annexin V Apoptosis Detection Kit (Yeasen Biotech). Following mild trypsin digestion, cells were doubly stained with FITC-Annexin V and propidium iodide as per the manufacturer’s protocol. Gallios flow cytometry (Beckman Coulter), coupled with Kaluza acquisition software, was employed to analyze at least 10,000 events per sample, distinguishing living, dead, early apoptotic, and apoptotic cells. For comparative purposes, the combined proportion of early apoptotic and apoptotic cells served as the primary outcome measure.

### Statistical analysis

Data are presented as mean ± SEM. Comparisons between groups were performed using t-tests or one-way ANOVA, followed by *post hoc* Bonferroni or Tukey tests, as appropriate. Statistical analyses were conducted using GraphPad Prism 8.0 (GraphPad Software Inc., San Diego, CA, USA) and SPSS 25.0 (SPSS Inc., Chicago, IL, USA) software.

## Results

### HFD exacerbates post-myocardial infarction cardiac injury

To assess the impact of a HFD on cardiac injury following MI, WT mice were maintained on either a normal diet (ND) or HFD for 8 weeks. After this period, the HFD-fed mice underwent left coronary artery ligation and were treated for an additional 4 weeks. To evaluate the effect of HFD on post-MI pathological remodeling, we analyzed cardiac function using echocardiography 4 weeks post-MI in both ND and HFD mice. The HFD-fed mice subjected to MI exhibited significantly worsened cardiac remodeling and heart failure (HF).

**Figure 1:**
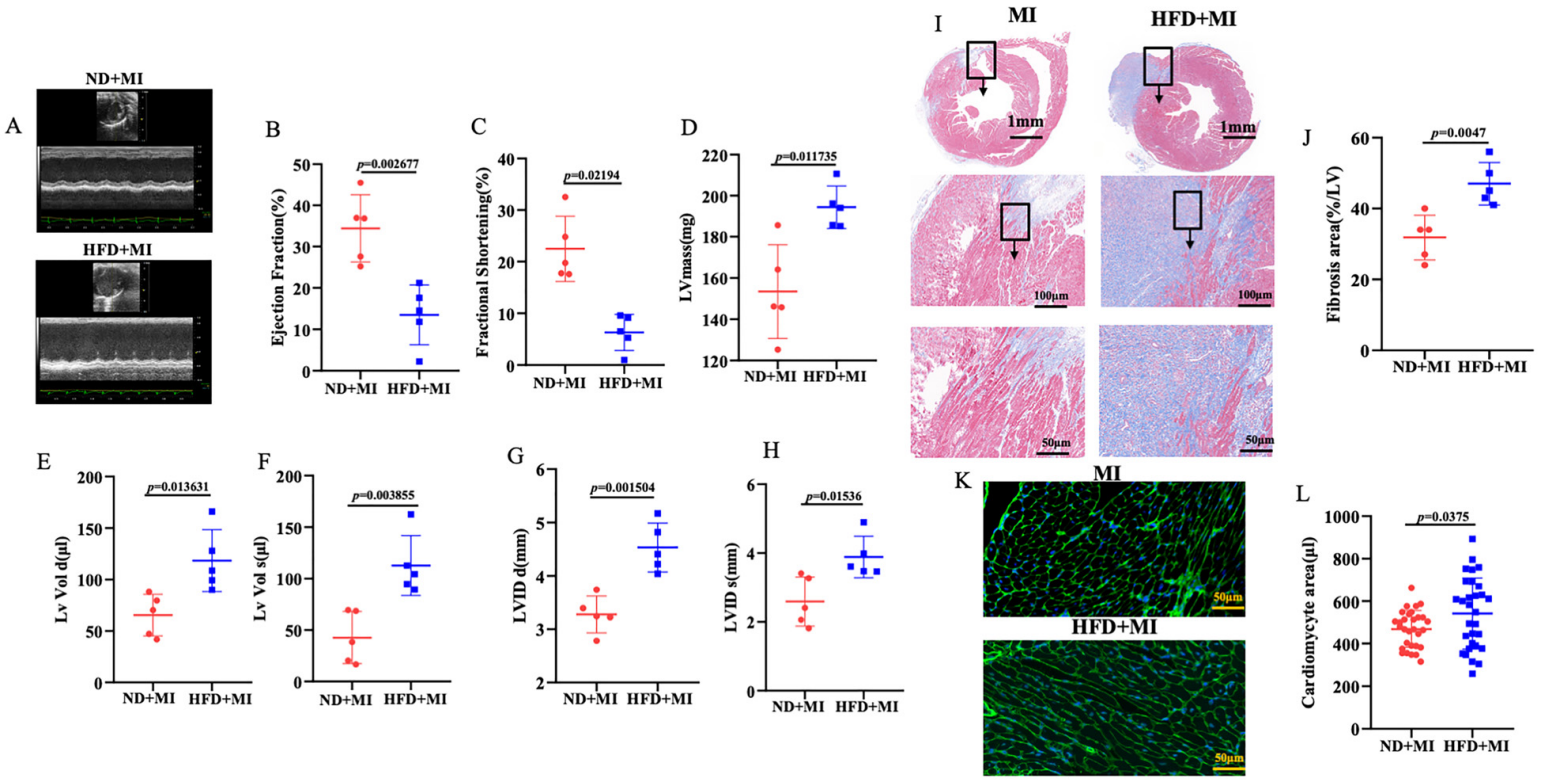
Diabetes increased post-MI cardiac injury. **(A)** Cardiac function was assessed using echocardiography, with two representative sets of left ventricular M-mode echocardiography images displayed. **(B-H)** Quantification of EF, FS, LVmass, LVIDd, LVIDs, LV Vol-d, and LV Vol-s was derived from the echocardiographic analysis. **(I, J)** Analysis of interstitial fibrosis. **(K, L)** Wheat germ agglutinin (WGA, scale bar=50μm) staining were performed to evaluate the cardiac hypertrophic growth. n = 5/group. EF, ejection fraction; FS, fractional shortening; LVmass, Left Ventricular Mass; LVIDd, Left Ventricular Internal Diastolic Diameter, LVIDs, Left Ventricular Internal Systolic Diameter; LV Vol-d, Left Ventricular Diastolic Volume; LV Vol-s, Left Ventricular Systolic Volume.

As illustrated in **Figures 1A–H**, diabetes exacerbates post-MI cardiac remodeling, as evidenced by reductions in ejection fraction (EF) and fractional shortening (FS) within the 4 weeks following MI. Meanwhile, left ventricular mass (LV mass), left ventricular volume (LV VOL), and left ventricular internal diameter (LVID) were significantly increased (p < 0.05). To provide further evidence that HFD exacerbates post-MI cardiac injury through increased hypertrophy and fibrosis, Masson staining and wheat germ agglutinin (WGA) staining were performed. The results demonstrated that HFD significantly increased interstitial fibrosis and cardiomyocyte cross-sectional area following MI (**Figures 1I–L**). Collectively, these data indicate that HFD is a critical factor contributing to increased cardiac injury after MI.

### HFD modulates post-myocardial infarction gene expression profile

**Figure 2:**
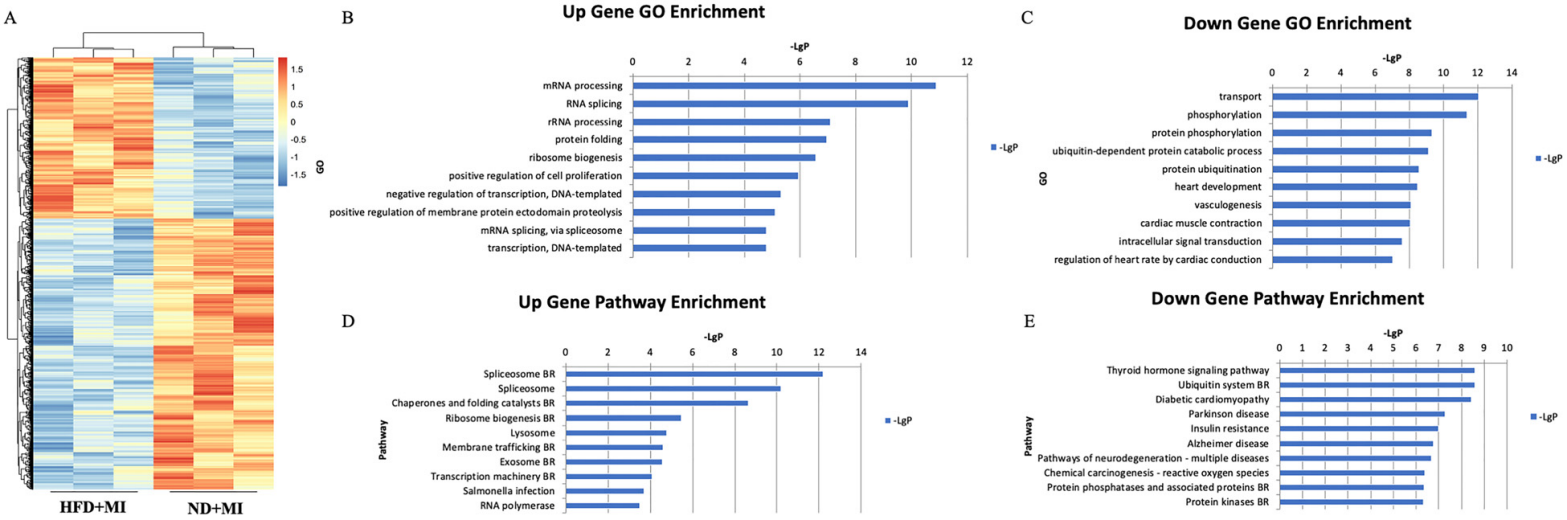
mRNA expression profile in cardiac tissues from MI mice with diabetes. **(A)** Differential expression of mRNA; **(B)** Upregulated genes. **(C)** Downregulated genes. **(D)** Significantly upregulated pathways. **(E)** Significantly downregulated pathways. The y-axis represents the GO category, and the x-axis represents the negative log-transformed p-value (-lgP), where a larger -lgP value indicates a smaller p-value.

To investigate the differences in mRNA expression patterns in heart tissue between HFD-fed and ND-fed mice post- MI, we performed transcriptome sequencing analysis for each group. The results revealed that HFD was associated with 2,316 differentially expressed genes compared to the ND-fed post-MI group, of which 865 genes were upregulated and 1,451 genes were downregulated (**Figure 2A**).

Top ten upregulated Gene Ontology (GO) terms associated with HFD included mRNA processing, RNA splicing, ribosomal RNA (rRNA) processing, protein folding, ribosome biogenesis, positive regulation of cell proliferation, negative regulation of transcription, DNA-templated transcription, positive regulation of membrane protein ectodomain proteolysis, and mRNA splicing via the spliceosome (**Figure 2B**). Conversely, the ten major downregulated GO terms related to transport, phosphorylation, protein phosphorylation, ubiquitin-dependent protein catabolism, protein ubiquitination, heart development, vasculogenesis, cardiac muscle contraction, intracellular signal transduction, and regulation of heart rate by cardiac conduction (**Figure 2C**).

According to the KEGG database, the top ten significantly upregulated pathway interactions in the HFD+MI group compared to the ND+MI group included the spliceosome, chaperones and folding catalysts, ribosome biogenesis, lysosome, membrane trafficking, exosome, transcription machinery, Salmonella infection, and RNA polymerase pathways (**Figure 2D**). In contrast, the top ten significantly downregulated pathways encompassed the thyroid hormone signaling pathway, ubiquitin system, diabetic cardiomyopathy, Parkinson’s disease, insulin resistance, Alzheimer’s disease, pathways of neurodegeneration (multiple diseases), chemical carcinogenesis (reactive oxygen species), and protein phosphatases and associated proteins (**Figure 2E**).

### Generation of global signal transduction network and pathway network

**Figure 3:**
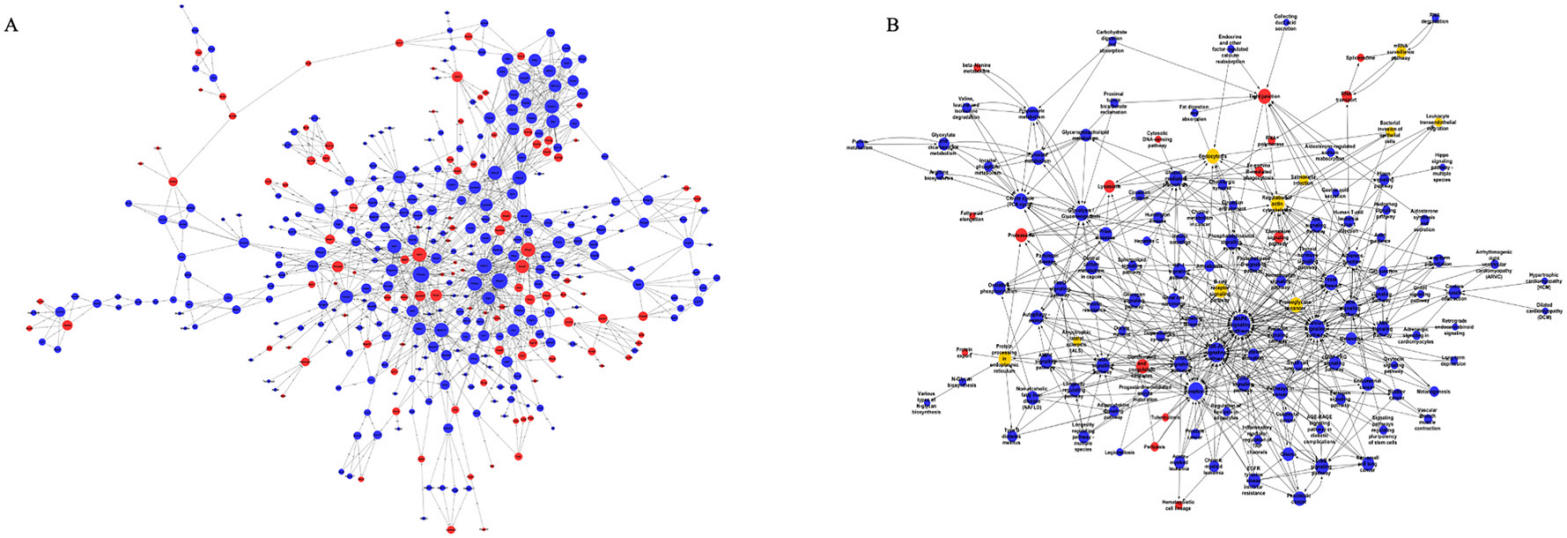
Global Signal Transduction Network and Pathway Network. **(A)** a co-expression network for differentially expressed mRNAs, constructed based on the results of mRNA sequencing. **(B)** a pathway network derived from KEGG-enriched pathways. The relationships between genes/pathways are represented by edges. And the size of each node indicates the correlation strength.

We established a co-expression network for differentially expressed mRNAs based on the results of mRNA sequencing, which enabled us to identify potential linkages between genes in the heart tissue of HFD+MI mice. Utilizing graph theory methodologies, we assessed the regulatory status of mRNAs, with the degree of each mRNA in the network serving as the evaluation criterion. Key mRNAs were identified as those with the highest degrees of connectivity within the network (**Figure 3A**). Additionally, we constructed a pathway network based on KEGG-enriched pathways. The findings highlight the core mRNAs and pathways that are most likely to contribute to post-myocardial infarction cardiac injury under diabetic conditions (**Figure 3B**).

### Heart tissue lncRNA expression profile and lncRNA-gene network in HFD-treated myocardial infarction group

**Figure 4:**
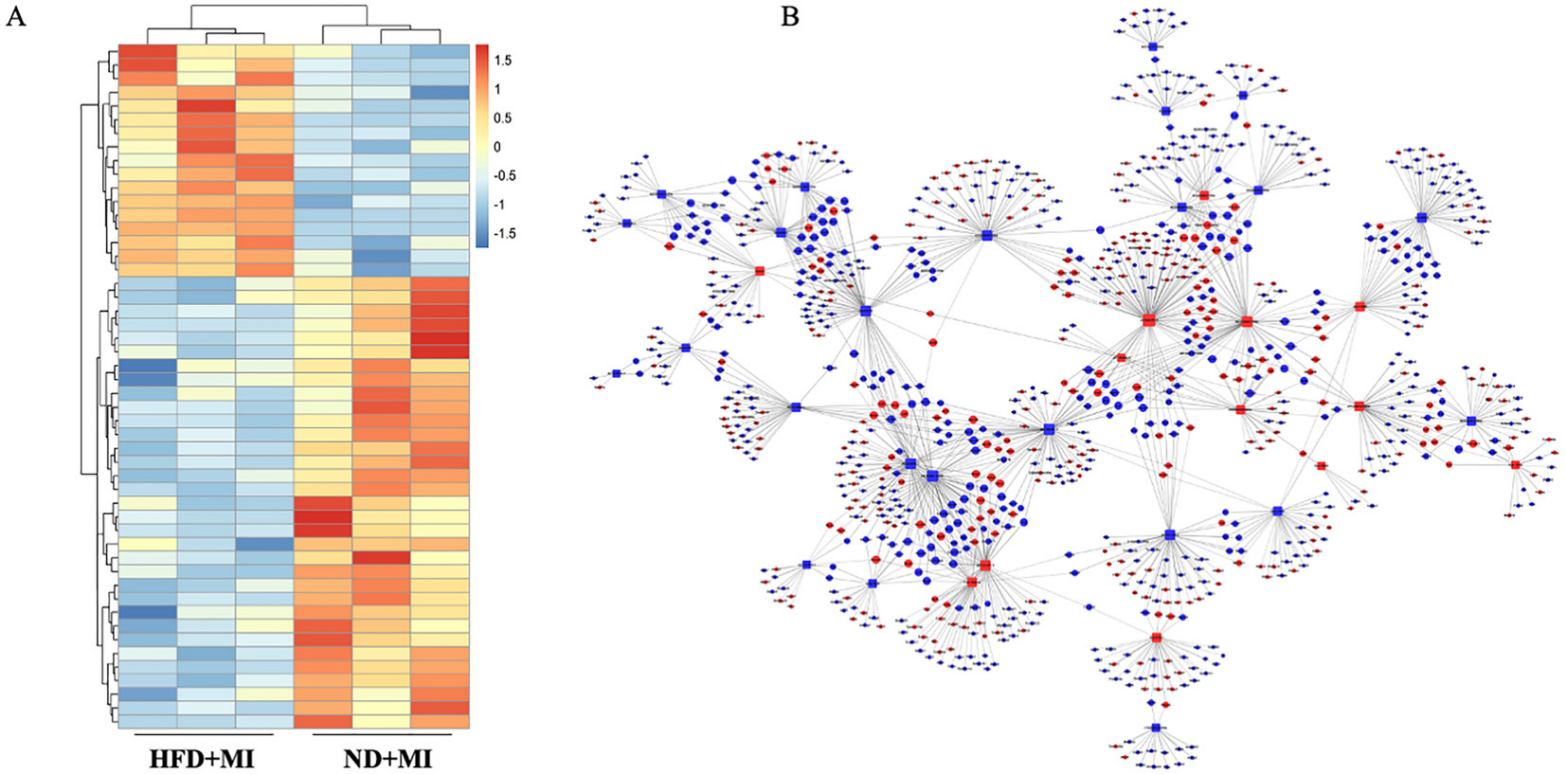
Hierarchical cluster reveals the relatedness of differentially expressed LncRNAs. **(A)** Heatmap showing the relative abundance of significantly altered lncRNAs in the ND+MI and HFD+MI groups. **(B)** LncRNA-mRNA expression correlation network analysis of core lncRNAs and their correlated mRNAs regulated by post-MI with diabetes. Square nodes represent lncRNAs, while round nodes represent mRNAs. The lines between nodes indicate a correlation, with a solid line representing positive correlation and a dotted line representing a negative correlation. The size of each node represents the correlation strength.

We further evaluated the impact of a HFD on lncRNA expression profiles during MI. Heart tissue samples from each group were collected for lncRNA sequencing analysis. A total of 50 differentially expressed lncRNAs (fold change ≥ 1.5, p-value < 0.05) were identified in the HFD+MI group compared to the ND+MI group (**Figure 4A**). The relative abundances of these lncRNAs, influenced by MI under diabetic conditions, are illustrated in the accompanying figure. Notably, the differentially expressed lncRNAs exhibited distinct expression patterns across the groups. Subsequently, we established a co-expression network for lncRNAs and mRNAs based on microarray results (**Figure 4B**). In this network, the degree of each lncRNA was defined as the number of mRNAs it regulates, while the degree of each mRNA was determined by the number of lncRNAs that regulate it. Key lncRNAs and mRNAs within the network were characterized by their higher degrees of connectivity. AC165273.2, 2310039LI5Rik, and AC160401.1. were identified in the network map with degrees greater than 75. Therefore, we propose that these three core lncRNAs play critical roles in the HFD+MI groups compared to ND+MI groups, warranting their selection for further analysis.

### Verification of key target genes in STEMI patients with or without diabetes

**Figure 5:**
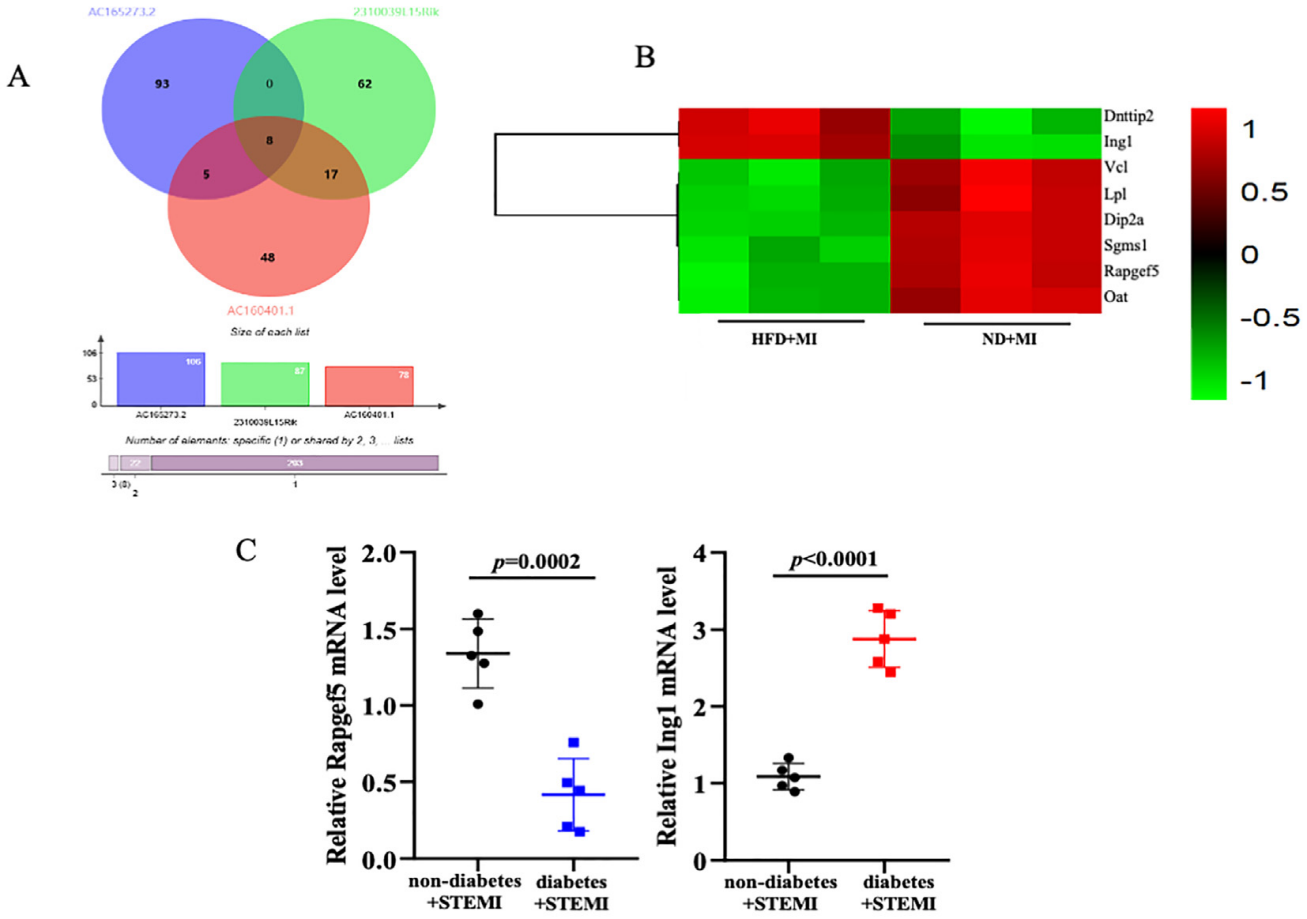
Verifying the target genes. **(A, B)** The overlapped target genes of AC165273.2, 2310039LI5Rik, and AC160401.1 are presented using venn diagram and heatmap. **(C)** The mRNA levels of target genes in the cardiac tissue of STEMI patients with diabetes were analyzed. N=5 independent experiments/group. Data were analyzed by t-test (GraphPad Prism 9.0).

Given the challenges associated with validating lncRNAs, we focused on verifying their target genes. A venn diagram illustrates the identification of a cohesive cluster of 8 target genes (Dnttip2, Ing1, Vcl, Lpl, Dip2a, Sgms1, Rapgef5, and Oat), as the shared intersections among AC165273.2, 2310039LI5Rik, and AC160401.1 (**Figures 5A, B**). Given the high degree of congruence between our meticulously crafted animal model and the clinical manifestations observed in patients with STEMI, we have strategically procured heart tissues from STEMI patients to further verify the expression of these eight target genes in patients with STEMI affected by diabetes. A total of 10 subjects (5 with diabetes+STEMI and 5 without diabetes+STEMI) were enrolled in this study. Demographic data, along with baseline clinical and biochemical characteristics, are summarized in **Supplementary Table S1**. Statistical analysis revealed that fasting blood glucose (FBG) levels were significantly elevated in diabetic patients. RNA was extracted from the cardiac tissue samples, and quantitative PCR (qPCR) was performed to validate the expression of these eight target genes. Among these genes, mRNA levels of Rapgef5 were significantly inhibited by diabetic conditions in STEMI patients. Conversely, Ing1 expression was markedly upregulated in the cardiac tissue of STEMI patients with diabetes compared to those without diabetes (**Figure 5C**). Collectively, these results, as presented in the accompanying figure, demonstrate that diabetes plays a crucial role in modulating gene expression in STEMI, which in turn influences the progression of cardiac injury.

### Both Rapgef5 and Ing1 are involved in proliferation and apoptosis of cardiomyocytes induced by HG/HL with hypoxia/reoxygenation

**Figure 6:**
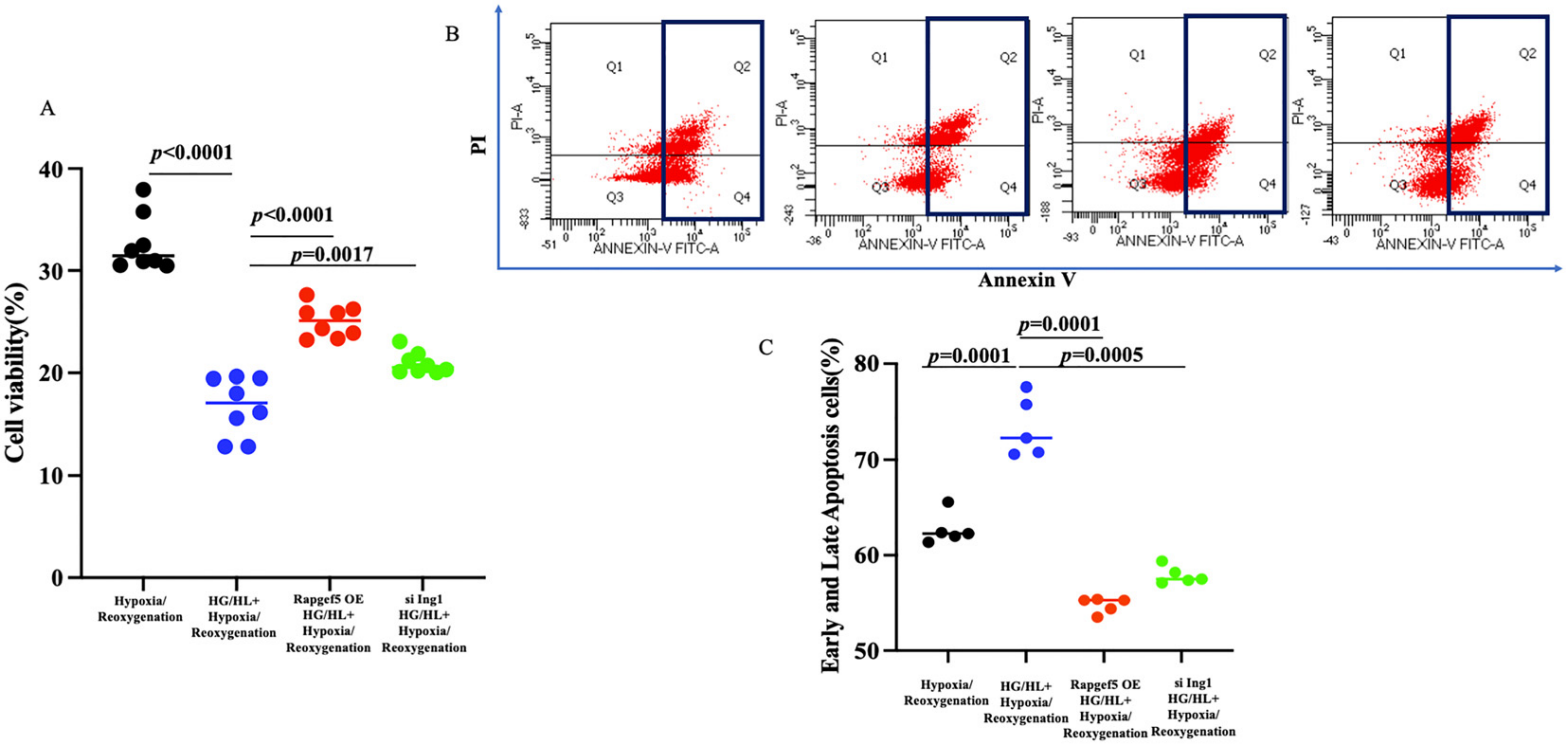
Both Rapgef5 and Ing1 are involved in proliferation and apoptosis of AC16 cells induced by HG/HL with hypoxia/reoxygenation. **(A)** CCK8 assay demonstrated that Rapgef5 overexpression and Ing1 knockdown promoted AC16 cells growth and viability. **(B, C)** Apoptosis in AC16 cells were determined by Annexin V/PI staining. N=5~8 independent experiments/group. Data were analyzed by two-way analysis of variance (GraphPad Prism 9.0).

Compared to the STEMI cohort, myocardial infarction (MI) patients with diabetes exhibited reduced expression of Rapgef5 and elevated expression of Ing1. These findings suggest that Rapgef5 may serve as a protective factor mitigating the development of diabetic MI, whereas Ing1 appears to contribute to the exacerbation of STEMI in diabetic patients. To elucidate the mechanistic roles of Rapgef5 and Ing1 in cardiomyocytes under diabetic myocardial infarction conditions, we investigated the effects of Rapgef5 overexpression and Ing1 knockdown in human cardiomyocytes (AC16) subjected to high glucose and high lipid (HG/HL) conditions in conjunction with hypoxia/reoxygenation. Cell viability was quantified using the CCK8 assay, while apoptosis was analyzed through Annexin V/PI staining followed by flow cytometry. As illustrated in **Figure 6A**, both Rapgef5 overexpression and Ing1 knockdown significantly enhanced cardiomyocyte proliferation and restored cell viability compromised by HG/HL combined with hypoxia/reoxygenation. Furthermore, as depicted in **Figures 6B, C**, apoptotic processes induced under these conditions were markedly attenuated by the overexpression of Rapgef5 or knockdown of Ing1. Collectively, these findings underscore the critical roles of Rapgef5 and Ing1 in cardiomyocyte survival and proliferation. Specifically, upregulating Rapgef5 and downregulating Ing1 offer promising strategies for inhibiting apoptosis and enhancing cell viability in the context of diabetic myocardial infarction.

## Discussion

In recent years, the incidence of diabetes has risen significantly, becoming one of the most prevalent diseases impacting the health and quality of life of the Chinese population (13). Research indicates that diabetes is a major risk factor for MI (6), and an increasing number of studies highlight the pivotal roles of lncRNAs and mRNAs in the occurrence and progression of diabetic complications (8). However, the specific biomolecular changes in cardiac tissues during myocardial infarction under diabetic conditions remain poorly understood. Our study elucidates the molecular alterations of lncRNAs and mRNAs in cardiac tissues affected by both diabetes and myocardial infarction, thereby exploring the potential of modern molecular indicators in predicting myocardial infarction in diabetic patients.

Cardiac remodeling is a physiological response to cardiac injury or hemodynamic stress, leading to changes in the size, shape, and function of the heart, driven by alterations in molecular and gene expression (14). This process typically follows MI and occurs in conditions such as chronic myocarditis, dilated cardiomyopathy, and heart failure (15). During the onset of these diseases, the heart undergoes remodeling as a compensatory mechanism. Therefore, early intervention to inhibit cardiac remodeling represents an effective strategy for delaying the onset of heart failure in patients with cardiovascular diseases. Numerous clinical studies have demonstrated that elevated levels of glucose and lipids can exacerbate post-MI cardiac remodeling (16). Our research also indicates that diabetes significantly exacerbates post-MI cardiac remodeling. In our study, diabetic mice with MI exhibit enlarged hearts, increased infarct sizes, and impaired cardiac function. Furthermore, results from Masson’s trichrome staining and WGA staining reveal increased fibrosis and more pronounced myocardial hypertrophy in the hearts of diabetic mice with MI.

High-throughput sequencing technology, commonly referred to as next-generation sequencing, enables the simultaneous sequencing of tens of thousands to millions of nucleic acid molecules in a single run (17). Recently, the application of lncRNAs and mRNAs in disease diagnosis and treatment has emerged as a prominent area of research. This technology facilitates the identification of expression profiles of lncRNAs and mRNAs in various tissues or organisms, thereby allowing for the analysis of the relationship between differential lncRNA and mRNA expression and disease states (18). LncRNAs and mRNAs influence the onset and progression of metabolic diseases through diverse mechanisms, with techniques for their interference and overexpression playing pivotal roles in therapeutic applications (18). Previous studies have highlighted the role of disordered expression of lncRNA-H19 in the development of cardiovascular diseases (12) and demonstrated that lncRNA MALAT1 regulates mitochondrial dynamics post-myocardial infarction while inhibiting myocardial apoptosis through the miR-26B-5p/Mfn1 axis (10). Furthermore, lncRNA TUG1 has been identified as a potential therapeutic target following myocardial infarction (19). In our study, we employed lncRNA sequencing analysis and mRNA sequencing technology to elucidate molecular changes in cardiac tissue following myocardial infarction. Our results identified a total of 50 differentially expressed lncRNAs and 2,316 differentially expressed mRNAs in diabetic hearts post-MI. Gene Ontology (GO) annotation, a classical method for functional analysis of differentially expressed genes, indicated that the most prominent gene function categories affected by diabetes, according to our sequencing data, were primarily related to gene splicing processes. Consistent with prior research indicating that high glucose and lipid levels can impact gene expression, synthesis, and protein cleavage, our findings suggest that diabetes may disrupt gene synthesis mechanisms.

The lncRNA gene regulatory network diagram is constructed based on the similarity of intergenic expression data, connecting genes with similar expression profiles to form a cohesive network (20). By establishing this gene co-expression network, we can explore the regulatory effects of lncRNAs on genes in greater depth, enabling the identification of core lncRNAs and their corresponding target genes. The global signal regulatory map of lncRNAs on genes highlights three pivotal lncRNAs—AC165273.2, 2310039L15Rik, and AC160401.1—situated at the network’s hub. To clarify the significance of core lncRNA, we collected heart tissue samples from patients with STEMI for further research. Based on blood glucose and glycated hemoglobin levels, the patients were divided into a diabetic group and a non-diabetic group. The blood glucose and glycated hemoglobin levels in the diabetic group and in the non-diabetic group showed statistically significant differences. Additionally, there was no significant difference in ejection fraction (EF), an indicator of cardiac function, between the diabetic and non-diabetic groups in the clinical samples collected. Based on the above baseline data, it is suggested that our sample collection was reasonable. Given the scarcity of annotation information with validating lncRNAs in human samples, we selected eight target genes from the intersection of these three lncRNAs, which exhibited high eigenvalues, for quantitative PCR (qPCR) validation. Among these, two data points aligned with the RNA sequencing results, revealing downregulation of the Rapgef5 genes and upregulation of the Ing1 gene in cardiac tissues of patients with diabetes mellitus complicated by myocardial infarction. Rapgef5 has been observed to be significantly upregulated in cancerous tissues and cell lines. Functional studies have demonstrated that its downregulation suppresses cancer cell proliferation, migration, and invasion, while promoting apoptotic processes (21). Similarly, Ing1 has been characterized as a tumor suppressor, with knockout mice exhibiting stunted growth, increased sensitivity to ionizing radiation, and the development of large clear B-cell lymphomas. Consistent with its tumor suppressive role, Ing1 protein levels are markedly lower in primary breast tumors and established breast cancer cell lines compared to normal tissues and cells (22). Despite these advances, the involvement of Rapgef5 and Ing1 in the pathogenesis and progression of diabetic myocardial infarction remains unexamined, highlighting a critical gap in the current understanding of these factors. In the present study, we provide evidence that upregulation of Rapgef5 and downregulation of Ing1 effectively mitigate cardiomyocyte apoptosis and promote proliferation under conditions of high glucose and high lipid (HG/HL) exposure coupled with hypoxia/reoxygenation. These findings suggest novel regulatory roles for Rapgef5 and Ing1 in the cellular response to diabetic myocardial infarction. Based on the available search results, the mechanistic roles of Rapgef5 and Ing1 in diabetic myocardial infarction (MI) remain underexplored. Although numerous studies focus on pathways related to cardiovascular injury and repair, none specifically investigate the involvement of these two molecules in the context of diabetic MI. The following is an analysis of potential mechanisms inferred from related findings, as well as the existing gaps in the current literature. Rapgef5 is a guanine nucleotide exchange factor (GEF) that activates Rap1, a small GTPase involved in cell adhesion, angiogenesis, and inflammation (23, 24). Evidence indicates that hyperglycemia and insulin resistance disrupt endothelial function and promote oxidative stress in diabetic conditions (25). Theoretically, Rapgef5-mediated Rap1 activation could enhance endothelial integrity and reduce inflammation, as Rap1 stabilizes adherens junctions and suppress inflammation signaling. Ing1 modulates p53 activity, a pivotal regulator of cardiomyocyte apoptosis (26, 27). In diabetic MI, persistent hyperglycemia amplifies cardiomyocyte apoptosis (28). however, no studies here invetigated Ing1’s interaction with p53 or other apoptotic pathways in this context. Ing1 also participates in histone acetylation/deacetylation, influencing inflammatory gene expression (29). Diabetic MI involves epigenetic dysregulation (30), yet Ing1’s contribution remains uninvestigated. In summary, while the provided literature does not directly address Rapgef5 and Ing1 in diabetic MI, their known functions in apoptosis, inflammation, and epigenetic regulation suggest plausible mechanistic links. Future studies should integrate diabetic models with molecular profiling to unravel these pathways.

In conclusion, we got several innovative discoveries. First, our study determined that an LncRNA and mRNA expression profile is related to remarkable changes in heart tissues from HFD-Treated myocardial infarction group. Second, our data supports that Rapgef5 and Ing1 may play a crucial role in HG/HL with hypoxia/reoxygenation-induced proliferation and apoptosis of cardiomyocytes. Further studies are needed to confirm the mechanisms by which RAPGEF5 and Ing1 exert their effects.

## Data Availability

The original contributions presented in the study are included in the article/**Supplementary Material**. Further inquiries can be directed to the corresponding authors.
