# Supplementary material for: Identification of novel target genes in exaggerated cardiac remodeling following myocardial infarction in diabetes
Source: Front Endocrinol (Lausanne). 2025 Mar 14;16:1536639. doi: 10.3389/fendo.2025.1536639 (PMC11949792; doi:10.3389/fendo.2025.1536639)
Supplement: Supplementary file 1 [file Table1.docx]

**Supplemental Table 1. The baseline characteristics of study subjects**

| Variable | STEMI group(n=5) | STEMI with diabetes(n=5) | *P* value |
| --- | --- | --- | --- |
| Age (years) | 64(55-70) | 57.5(48.75-68.25) | 0.123 |
| Glucose (mmol/L) | 5.57±0.74 | 8.34±0.91 | <0.0001 |
| HbA_1C_ (%) | 5.72±0.3 | 6.75±0.2 | <0.0001 |
| TCH (mmol/L) | 4.08±1.03 | 4.03±0.99 | 0.7358 |
| TG (mmol/L) | 1.63±0.77 | 1.57±0.65 | 0.3457 |
| LDL-C(mmol/L) | 2.54±1.12 | 2.48±0.97 | 0.8434 |
| HDL-C(mmol/L) | 1.03±0.14 | 1.07±0.19 | 0.3875 |
| C-reactive protein(mg/L) | 3.52±5.33 | 5.37±7.38 | 0.0483 |
| EF (%) | 54.72±8.96 | 54.95±9.06 | 0.0938 |

***Abbreviations:*** HbA1C: glycated hemoglobin; TCH: total cholesterol; TG: Triglyceride; LDL-C: low-density lipoprotein; HDL-C: high-density lipoprotein; EF: ejection fraction

**Supplemental Table 2: Real Time PCR Primers**

| Gene Profile | Species | Genes | Forward primer (5’-3’) | Reverse primer (5’-3’) |
| --- | --- | --- | --- | --- |
| House  keeper | Homo sapiens | GAPDH | AGGTCGGTGTGAACGGATTTG | TGTAGACCATGTAGTTGAGGTCA |
| Gene | Homo sapiens | Lpl | CCGAGAGTGAGAACATCCCATTCA | CCTTTCTGCAAATGAGACACTTTCTC |
| Gene | Homo sapiens | Dip2a | GGTGAACCTGTCATGTGTGC | CAGGTCCTTGAAGAGCTTGG |
| Gene | Homo sapiens | Vcl | CTCGTCCGGGTTGGAAAAGAG | AGTAAGGGTCTGACTGAAGCAT |
| Gene | Homo sapiens | Oat | TTGCGCTTCATAGACGCC | AAAACCACAGATCTGACAGCG |
| Gene | Homo sapiens | Dnttip2 | ATGAGAGCCAGCATGGACC | ACTTCCTTCGGTTGTATCTTCTGA |
| Gene | Homo sapiens | Sgms1 | ACCATAAGAGAAAGTAGTGACAAGTG | GCCAGGACTTGATCAACCTAACC |
| Gene | Homo sapiens | Rapgef5 | CAGCAAAGGTCGATGAGGAT | TCATTGCATCTCTGGAGCAG |
| Gene | Homo sapiens | Ing1 | AAGTGGTACTGTCCCAAGTGCC | GCCCTCTCTTTTTTGGATTTCTCC |
